# Recent insight and future techniques to enhance rumen fermentation in dairy goats

**DOI:** 10.5713/ajas.19.0323

**Published:** 2019-07-01

**Authors:** Lovelia L. Mamuad, Sung Sill Lee, Sang Suk Lee

**Affiliations:** 1 Department of Animal Science and Technology, Sunchon National University, Suncheon, Jeonnam 57922, Korea; 2 Division of Applied Life Science (BK21 Program) and Institute of Agriculture & Life Science (IALS), Jinju 52828, Korea

**Keywords:** Dairy Goats, Dietary Interventions, Omics Techniques, Probiotics, Prebiotics

## Abstract

Recent development of novel techniques in systems biology have been used to improve and manipulate the rumen microbial ecosystem and gain a deeper understanding of its physiological and microbiological interactions and relationships. This provided a deeper insight and understanding of the relationship and interactions between the rumen microbiome and the host animal. New high-throughput techniques have revealed that the dominance of Proteobacteria in the neonatal gut might be derived from the maternal placenta through fetal swallowing of amniotic fluid in utero, which gradually decreases in the reticulum, omasum, and abomasum with increasing age after birth. Multi “omics” technologies have also enhanced rumen fermentation and production efficiency of dairy goats using dietary interventions through greater knowledge of the links between nutrition, metabolism, and the rumen microbiome and their effect in the environment. For example, supplementation of dietary lipid, such as linseed, affects rumen fermentation by favoring the accumulation of α-linolenic acid biohydrogenation with a high correlation to the relative abundance of Fibrobacteriaceae. This provides greater resolution of the interlinkages among nutritional strategies, rumen microbes, and metabolism of the host animal that can set the foundation for new advancements in ruminant nutrition using multi ‘omics’ technologies.

## INTRODUCTION

The rumen has a diverse and complex microbial ecosystem that converts ingested plant biomass into proteins, volatile fatty acids (VFAs), and vitamins to be used by the host animal. Studying the relationships and interactions of microbes in the rumen is difficult as different microbes have different substrates, requirements, and digestive roles. However, understanding the relationships and interactions between microbes is important for evaluating their effects on the environment or in the community they inhabit, as they play a pivotal role in animal health, performance, milk [1], and meat quality and composition. High-throughput novel “omics” systems biology techniques, such as genomics, transcriptomics, proteomics, and metabolomics, enable researchers to analyze biological molecules and profile microorganisms in detail to understand the rumen microbial community structure, metabolic potential, and activity.

The goat is smaller than other ruminants, such as cattle; hence, they are categorized as small ruminants. The goat rumen also has a different motility pattern than that of cattle as it has reticular motility during rest by a biphasic contraction and longer duration of reticular regurgitation contraction [2]. Goat rumen improvement could be achieved via elevated production of microbial crude protein in the rumen microbiota through dietary interventions and supplementation with prebiotics, probiotics, and/or enzymes. However, even with the vast increase in livestock products, animal scientists and nutritionists are still exploring ways to improve ruminant nutrition to meet the demands of a growing human population coupled with increasing economic wealth. Animal scientists and nutritionists use several approaches to meet these demands. One of these approaches is modulating rumen nutrition through dietary interventions, the use of prebiotics and probiotics, and recent omics technologies, which have helped to elucidate insight into the microbiota dynamics of the goat rumen, as well as the other stomach compartments, such as reticulum, omasum, and abomasum. The objective of this review is to summarize recent insights and the potential of future techniques in enhancing rumen fermentation in dairy goats.

## THE GOAT RUMEN AND MICROBIAL COMMUNITIES

The goat rumen varies in pH, microbiome, fermentation products, and metabolites depending on the diet ingested by the animal. On a high forage diet study of Lee et al [3] with commercial concentrate mix and rice straw in the ratio of 9:1, the goat rumen had a pH of 6.25, while on a high grain diet (animals were only fed with concentrate for 1 week) it had a pH of 5.90 [4]. However, the ruminal pH of lactating Nubian goats fed with 500 g Egyptian berseem clover and 500 g concentrate feed mixture [5], 400 g Egyptian berseem clover and 600 g concentrate feed mixture [6] and Saanen [7] goats fed with 50:50 forage to concentrate ratio was 6.00 to 6.13 and 6.17, respectively. Usually, ruminal pH is correlated with the concentrations of VFAs. In the study of Kholif et al [5] and Kholif et al [6], lactating Nubian goat rumens have 26.9 g/L [5] to 29.2 g/L [6] ammonia nitrogen and 82.0 mmol/L VFA with acetate, propionate, and butyrate concentrations of 59.0, 26.4, and 2.25 mmol/100 mmol VFA [5], respectively. The rumen of Korean native goats on high forage and concentrate diets have comparable ammonia nitrogen and total VFA, and only differ in acetate production, which is higher on a high forage diet [4].

Goat rumen microbiota composition is usually dominated by Bacteroidetes followed by Firmicutes with a very low abundance of Fibrobacteres [3,7–9]. Contrastingly, Cremonesi et al [1] reported that the rumen composition of Alpine dairy goats was dominated by Bacteroidetes with about 61.2% relative abundance, followed by Firmicutes, Proteobacteria, and Verrucomicrobia at 24.2%, 4.1%, and 3.3% relative abundances, respectively. Conversely, Bacteroidetes and Firmicutes were the predominant bacterial phyla while Clostridia and Bacteroides, were the dominant classes in Moxoto dairy female goats [10]. Meanwhile, the archaeal diversity of alpine dairy goats was dominated by Methanobacteriaceae and Methanomassiliicoccaceae with relative abundances of 36.8% and 37.1%, respectively [1], while Methanobrevibacter and Methanosphaera were found in Moxoto dairy female goats [10].

Operational taxonomic units of Bacteria and Archaea in Korean native goats are comparatively lower than those in Holstein cows and Hanwoo steers, which indicates that Korean native goats contain fewer bacterial and archaeal species [3]. On the contrary, a higher rumen microbial diversity in Korean native goats compared with Holstein and Hanwoo cows was reported by Lee et al [4] based on the Shannon diversity index and Inverse-Simpson index using T-RFLP analysis. Using high-throughput sequencing techniques, Henderson et al [11] reported that rumen protozoal community structure had a strong host individuality, wherein they were dominated by *Entodinium* and *Epidinium*. They also reported that the *Ophryoscolex* genus, which had a wider host distribution, was found in goats from 18 countries. In accordance, Lee et al [3] reported that goats have ~10^4^/mL copies of the 18S protozoa rRNA gene. In addition, Wang et al [9] explored the rumen microbiome of 55 male crossbred goats in 11 different age groups and found that the bacterial communities were mainly composed of Bacteroidetes, Firmicutes, and Proteobacteria, with Euryarchaeota and Thaumarchaeota in Archaea. *Fibrobacter succinogenes* had the highest relative abundance in the rumens of goats on a high forage diet with 2.187 compared to those on a high grain diet and to Hanwoo and Holstein cattle using quantitative real-time polymerase chain reaction [4].

Insight into the reticulum, omasum, and abomasum microbiota dynamics in pre-weaning goats has also been elucidated using metagenomics. Proteobacteria are facultative anaerobes, which dominate the neonatal gut owing to the unique abundance of oxygen. Studies have shown that Proteobacteria species may be derived from the maternal placenta through fetal swallowing of amniotic fluid in utero. It was also reported that Proteobacteria gradually decreased in the reticulum, omasum, and abomasum with the aging of the host [12]. Lerma-Reyes et al [13] reported that the reticulum, omasum, and abomasum were found to represent the primary, secondary, and third maturation stages of ruminant stomach compartments in early life. The primary stage occurs during the first two weeks after birth and exposure to foreign microorganisms, followed by the secondary stage (day 14 to 28 after birth), which is a period of microbial transition. Finally, the third stage (day 28 after birth and beyond) is the exogenous and endogenous microbial colonization stage [12].

## GOAT RUMINAL MICROBIOTA AND ITS PIVOTAL ROLE IN MILK COMPOSITION

Diet composition plays a pivotal role in the composition of ruminal microbiota, which defines ruminant products. The conjugated linoleic acid (CLA) in milk originates from either endogenous synthesis in the mammary gland from vaccenic acid or ruminal biohydrogenation of linoleic acid as an intermediate product [13]. Moreover, linseed induced fatty acid concentration shifts from C18:2n−6 to C18:3n−3 (α-linolenic acid [ALA]) with biohydrogenation. The fatty acid involved in the biohydrogenation pathway of ALA was reported to have the highly significant correlation with Fibrobacteriaceae [1], wherein *Fibrobacter succinogenes* is one of the most abundant saturated fatty acids characterizing principal species that produce C18:0 (stearic acid). *Fibrobacter succinogenes* also plays a role in the detoxification of the ruminal environment by using C18:3n−3 for its metabolism and producing C18:0 [1]. Dewanckele et al [14] added that the most important biohydrogenating bacteria are *Butyrivibrio* species, which have higher relative abundances when the reduction to C18:0 is inhibited.

Lipid supplementation of linseed increases the relative abundances of *Succinivibrio* spp. by 50% and *Fibrobacter* spp. by 75%. Consequently, linseed supplementation reduces the level of *Prevotella* spp. and dimethyl acetals (DMA), which are thought to play a role in plasmalogens [1]. This is because DMA is produced through acidic transesterification of fatty aldehydes, which are released from plasmalogens. The DMA are microbial membrane and cell wall structural lipids that regulate optimal fluidity of the membrane. Plasmalogens are one of the important classes of ruminal lipids that are present in large amounts in the membranes of anaerobic bacteria and are strictly related to bacterial metabolism. Plasmalogen content and composition of rumen microbes can be modified depending on their response to different environmental stimuli and microbial diversity [1].

## RUMEN MICROBIOTA AND ENTERIC METHANE MITIGATION

Methane is one of the products of normal fermentation of feedstuffs in the rumen. When methane is produced, the ruminant suffers a loss of ingested feed-derived energy of approximately 2% to 12%, depending on geographical location, feed quality, feed intake, feed composition, the processing of feed, and ruminant species [3,11,15]. Methane is produced by methanogens through enteric fermentation or methanogenesis. Methanogens are predominant among rumen Archaea, which belong to the phylum Euryarchaeota, and their populations differ depending on the species of the ruminant. Pseudomurein in Methanobrevibacter and Methanobacterium, heteropolysaccharide in Methanosarcina, and protein in Methanomicrobium form the cell wall of methanogens [15]. Methanogens use carbon dioxide and hydrogen, formate, methylamines, methanol, or acetate as the substrate to produce methane depending on their species and cell wall composition.

Pragna et al [16] reported that enteric methane can be mitigated through three mechanisms: targeting end products of digestion to propionate, providing alternate hydrogen sinks, and selectively inactivating rumen methanogens. These mechanisms decrease methane production through several strategies: dietary composition (by increasing starch, decreasing cell wall components, and grinding), lipids (fatty acids, oils, seeds, and tallow), defaunation (chemical and feed additives), methanogen vaccine, monensin, plant compounds (condensed tannins, saponins, and essential oils), or organic acids (fumarate or malate) [15]. Methane production was reduced by essential oils that directly inhibited methanogenic archaea [8].

Feed intake and feeding level are two of the factors that affect methane production in ruminants such as goats. Methane production in goats increases linearly with increased feeding level when expressed in g/d and g/kg body weight^0.75^ with 8.5, 10.3, and 11.2 g/d at feeding levels of 1.5%, 2.0%, and 2.5%, respectively [17]. However, the opposite is observed when methane production is expressed in g/kg dry matter intake, g/kg organic matter intake, and % gross energy intake. Zhou et al [18] reported that cattle with low feed efficiencies produced more methane gas and their methanogenic composition had more species variation (27 operational taxonomic units) and diversity (0.84 diversity index) than cattle with high feed efficiencies, which had less methane and lower methanogenic composition (22 operational taxonomic units and 0.42 diversity index). Zhou et al [18] also added that inefficient cattle have higher prevalence of *Methanosphaera stadtmanae* and *Methanobrevibacter* sp. strain AbM4 (1.92 and 2.26 times higher, respectively). Methane production can also be decreased by supplements of fumaric acid and tannins. Tannins can interfere with the membranes of rumen bacteria, and mitigate methane production, while fumaric acid stimulates the fumarate-utilizing bacteria, which increases propionate resulting in decreased methane production [19]. In addition, Mitsumori et al [20] reported that administration of 5 g of bromochloromethane-cyclodextrin/100 kg live weight of Japanese goats leads to 91% reduction in methane. Bromochloromethane-cyclodextrin showed dose-dependent methane mitigation in ruminants, shifting toward more propionic production and increased abundances of *Prevotella* spp., *Selenomonas* spp., and *Fibrobacter succinogenes* [20,21]. In addition, bromochloromethane-cyclodextrin administered to Japanese goats decreased methanogen diversity in the rumen with only 27% sequence data shared with control goats [21].

## FUTURE TECHNIQUES AND DIETARY INTERVENTIONS

**Figure 1:**
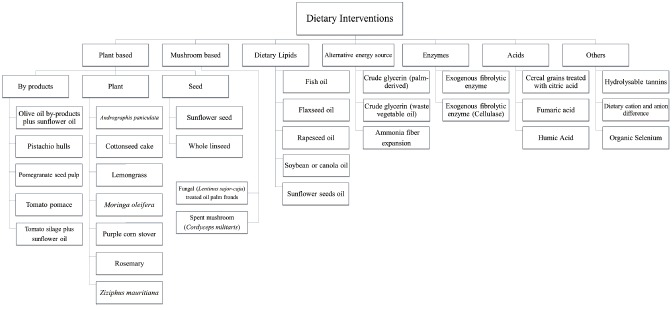
Recent dietary interventions in dairy goats.

Dietary interventions can be plant-based, mushroom-based, dietary lipids, alternative energy sources, enzymes, or acids (Figure 1). These dietary interventions can be used individually or in combination depending on the farmer’s choice. These dietary interventions affect animal performance, milk, and milk production.

### Increasing milk production and quality through dietary interventions

The use of rich sources of linoleic and ALA is essential in increasing the healthy properties of milk, because they favor the accumulation of CLA. Several studies have reported the positive effect of CLA in animals and humans. Consumption of CLA had been reported to have an effect on the immune system, atherosclerosis, and bone health [22]. The CLA in milk fat is also enhanced by lemongrass [23], *Moringa oleifera* [6,24], rosemary [23], sunflower seed linseed [1], by-products such as pistachio hulls [22], pomegranate seed pulp [22], and tomato pomace [22], and by oil-based supplements such as flaxseed oil [5], canola oil [13], soybean oil [13], and sunflower seeds oil [25] (Table 1). This improvement in the nutritional quality of milk can be attributed to the presence of *Butyrivibrio fibrisolvens*, which was found to increase after supplements of soybean oil [26]. Cremonesi et al [1] also reported that the *Butyrivibrio* group, which includes the genera *Butyrivibrio* and *Pseudobutyrivibrio*, were involved in the process of biohydrogenation.

Dietary interventions recently reported to be effective in increasing milk production include crude glycerin [27], exogenous fibrolytic enzyme (cellulase) [28], flaxseed oil [5], humic acid [29], lemongrass [23], *Moringa oleifera* [6,24], rosemary [23], sunflower seed and oil [25], and *Ziziphus mauritiana* [30] (Table 1). Also, goat milk quality can be improved by olive oil by-products plus sunflower oil [31], tomato silage plus sunflower oil [31], exogenous fibrolytic enzyme (cellulase) [28], humic acid [29], purple corn stover [32], and organic selenium [33].

Fatty acid profiles of ruminant products such as milk can be improved through changes in animal diet. Supplements of fish oil and plant oils in goat diets interact to modulate milk fat composition without decreasing milk fat content [34]. Hence, a diet with sunflower seed oil [25] was reported to increase milk fat content together with cottonseed cake [30], crude glycerin [27], *Grewia oppositifolia* [30], lemongrass [23], rosemary [23], and sunflower seed. The addition of lemongrass [23], rosemary [23], sunflower seed [25], flaxseed oil [5], rapeseed oil [35], sunflower seed oil [25], and hydrolyzable tannins [35] to the diet increases the total unsaturated fatty acids. Thoh et al [27] reported that supplementing goat diets with 5% crude glycerin increased milk fat via fermentation of crude glycerin into propionic acid, and increased daily intake and digestibility of lipids. Meanwhile, vaccenic (*t*11–18:1) contents in milk fat were also enhanced with dietary interventions such as pistachio hulls [22], pomegranate seed pulp [22], tomato pomace [22], and fish oil [34].

### Relations between rumen microbiota, body performance, and dietary intervention

Body performance can be improved by modulating the body system, increasing the feed intake, increasing or alleviating ruminal acidosis, improving digestibility, VFA profiles, and metabolites (Table 1). Body performance can be improved by improving animal energy balance through supplements and/or replacement of plant-based by-product, tomato silage with dietary lipids, sunflower oil [31], essential oil-cobalt [8], exogenous fibrolytic enzyme [36], and dietary cations and anions [37]. By partially replacing conventional forage with tomato silage plus sunflower oil [31], the body performance of dairy goats during mid-lactation was improved by increasing their body weight gain without compromising milk production and composition. Diets with a high non-fiber carbohydrate to neutral detergent fiber ratio and exogenous fibrolytic enzyme improved growth performance in Lezhi black goats by improving the average daily gain, feed conversion ratio, and nutrient utilization [36]. This could be due to increased microbial protein supply to the small intestine.

High-throughput sequencing and advanced techniques have been used to compare the effect of breed on the rumen microbiome and its associations with feed efficiency. In beef cattle, 8 bacterial phyla, including Bacteroidetes and Spirochaetes, and 55 taxa at the genus level such as *Prevotella* and *Treponema* were affected by breed [38]. Meanwhile, Shabat et al [39] and Wang et al [40] reported that the rumen bacterial and archaeal communities in alpha-diversity indices contributed to the variation in feed efficiency of cattle, wherein more complex and diverse microbial communities occurred in inefficient animals. Shabat et al [39] added that higher efficiency was tightly linked to lower richness of rumen microbiome gene content and taxa. Using metagenomic and metatranscriptomic analyses, *Chloroflexi*, *Blautia*, and a *Mogibacteriaceae*-affiliated unnamed genus were reported to be associated with feed efficiency in beef cattle rumen microbiome [38]. *Chloroflexi* was identified as related to host phenotype milk yield and diet adaptation, while *Blautia* was reported to provide energy to hosts from the fermentation of polysaccharides. Furthermore, efficient cows had a higher number of total short-chain fatty acid (10% higher) concentrations of propionate, butyrate, valerate, and isovalerate, and propionate to acetate than inefficient cows [39].

Dietary interventions that increase feed intake are *Moringa oleifera* [6,24], flaxseed oil [5], and exogenous fibrolytic enzyme (cellulase) [28], while *Andrographis paniculata* [41,42] and cereal grains treated with citric acid [43] are used to increase pH and alleviate ruminal acidosis. Cereal grains treated with organic acids increased ruminal resistant starch and relieved the risk of ruminal acidosis as well as reducing the inflammatory response [43]. In addition, fungal (*Lentinus sajor-caju*)-treated oil palm fronds [44], spent mushroom (*Cordyceps militaris*) [45], *Moringa oleifera* [6,24], lemongrass [23], rosemary [23], flaxseed oil [5], exogenous fibrolytic enzyme (cellulase) [28], and exogenous fibrolytic enzyme [36] were used to improve digestion in goats.

Anaerobic microbes in the rumen produce enzymes which the ruminants themselves can not. However, these enzymes are not enough to degrade most of the complex plant polysaccharides. Hence, exogenous fibrolytic enzyme supplements increase cellulase and xylanase activities, which increase fiber degradation. The exogenous fibrolytic enzyme helps in removing the structural barriers in feeds during digestion and thus improves the digestibility that allows cellulolytic bacteria to out-compete fungi for colonization in plant fibers. In addition, the improvement was due to improved energy substance metabolism, which also contributed to glycogenesis [34].

Meanwhile, the VFAs increased with a combination of essential oil and cobalt [8], fungal (*Lentinus sajor-caju*)-treated oil palm fronds [44], *Andrographis paniculata* [41,42], fumaric acid [19], and exogenous fibrolytic enzymes [36], while spent mushroom (*Cordyceps militaris*) was used to improve blood metabolites [45]. Moreover, *Andrographis paniculata* [41,42], and organic selenium [33] improved goat meat quality. Essential oils are plant-derived volatile aromatic compounds that have antibacterial, antiviral, antifungal, anti-nematode, insecticidal, and antioxidant properties. Cobalt is a co-factor of vitamin B12 that activates various enzymes. Supplements of combined essential oil and cobalt were reported to improve VFA production, which was positively associated with *Bacteroides* sp. and *Succinivibrio* sp. abundance [8]. In addition, a higher capacity for nitrogen utilization, xenobiotic biodegradation, and metabolism was observed in microbiota supplemented with a combination of essential oil and cobalt.

In tropical countries, heat accumulation and prolonged high ambient temperature affect animal performance and milk production due to reduced feed intake. When feed intake decreases, the intake of mineral elements also decreases. Hence, increasing dietary cation-anion difference (DCAD) is a necessity. Nguyen et al [37] reported that animals supplemented with high DCAD increased their total body water and apparent water balance through evaporative cooling, which slowed down the elevation in rectal temperature [37].

## THE SYMBIOSIS OF RUMEN MICROBIOTA, PROBIOTICS, AND PREBIOTICS

Ruminal microbiota play a pivotal role in animal nutrient utilization and synthesis for production and productivity. However, in the rumen, both synergistic and antagonistic interactions occur among microbes. These are essential in breaking down feed to produce VFAs that are then used by the animal as a source of energy. Probiotics are live microbes that stimulate beneficial microbes in the rumen while prebiotics are non-digestible sugars that provide substrates for beneficial microbes that induce their growth and activity. Probiotics and prebiotics are now widely used in livestock farming to benefit production and development, and address major public health concerns over antibiotic, antimicrobial, and hormonal growth promotors.

Probiotics have been shown to promote growth and health through increasing body weight gain, feed intake, and reducing diarrhea. Probiotic products may contain one or more selected strains, which are often gram-positive bacteria. The widely- used probiotics for ruminants are *Bacillus*, *Bifidobacterium*, *Enterococcus*, *Lactobacillus*, *Pediococcus*, *Streptococcus*, yeasts (*Saccharomyces cerevisiae*, *Kluyveromyces* sp.), and anaerobic fungus (*Aspergillus oryzae*). Aside from these widely known probiotics, there is continued research for new microbes that stimulate potentially beneficial rumen microbes. These are the cellulolytic bacteria, such as *Cellulomonas cellulase*, *Bacillus* sp., *Thermomonospora fusca*, *Acetobacter xylinum*, *Ruminococcus albus*, and *Clostridium cellulovorans* and fibrolysozyme or fibrolytic enzyme (fibrozyme), which is compounded from *Aspergillus niger*, *Trichoderma longibrachiatum*, fermentation extracts, and fermentation soluble [46].

In addition, important microbes involved in the pathway of rumen metabolism are also being explored. These are the fumarate reducing bacteria (*Mitsuokella jalaludinii* [47,48] and *Enterococcus faecium* [49], ruminal acetogens, *Acetitomaculum ruminis*, *Eubacterium limosum* strains ATCC 10825 and ATCC 8486, *Ruminococcus productus* ATCC 35244 [50], *Ruminococcus schinkii* sp. nov. [51], and *Proteiniphilum acetatigenes* [22]), the propionate-producing bacteria (*Coprococcus catus* [14], *Lactobacillus mucosae* [52,53], *Megasphaera elsdenii* [54], *Propionibacterium acidipropionici* [55], *Prevotella ruminicola* [56], *Selenomonas ruminantium* [57], and *Veillonella parvula* [58]), and the butyrate-producing bacteria (*Butyrivibrio fibrisolvens* [59]).

Fungi are one of the important microorganisms that have been used in farming owing to their multiple functions and beneficial effects. *Aspergillus oryzae* and *Saccharomyces cerevisiae* are some of the commercially available products. The function of probiotics exerts several effects, one of which is immunostimulation. The immunostimulant molecules in probiotic yeasts are β-glucans, chitins, mannans, and polyamines [60]. *Saccharomyces cerevisiae* is one of the well-known probiotics that produces vitamin B, organic acids, and amino acids [61]. The use of *Saccharomyces cerevisiae* as a ruminant feed additive could help the rumen bacteria to compete, or at least co-metabolize H_2_ with methanogens [62]. Moreover, *Saccharomyces cerevisiae* scavenges oxygen, which creates a more anaerobic environment in the rumen that enhances the hydrogenotrophic metabolism of the acetogenic strain by more than fivefold. This stimulates the utilization of hydrogen by the acetogenic strain and enhances acetogenesis [62]. In addition, supplements of yeast alleviate heat stress by reducing the absorption of endotoxins that effectively strengthen the antioxidant capacity of dairy goats [63]. Furthermore, the live *Debaryomyces hansenii* CBS 8339, a probiotic that is commonly found in marine environments, stimulates innate immune and antioxidant parameters and the expression of immune-related gene signaling pathways [60].

Prebiotics modulate the balance and activities of the rumen microbiota. Prebiotics contain specific nondigestible nutrients, which can be consumed raw or as functional foods and pharmaceutical preparations. Prebiotics include fructooligosaccharides, galactosyl-lactose, mannan oligosaccharides, pectin oligosaccharides, and xylooligosaccharides (Table 2).

## CONCLUSION

High-throughput technologies such as “omics” technologies combined with recent dietary interventions have provided a deeper insight into the relations and interactions between the rumen microbiome, metabolites, genes, and host health, performance, and efficiency. Further research to improve and understand the functions and interactions among the hosts, microbiome, and the feed is required.

## Figures and Tables

**Table 1:** List of recent dietary interventions and their main effects on dairy goats

Main effects on dairy goats	Dietary interventions	References
Body performance		
Improved body performance	Dietary cation and anion difference, exogenous fibrolytic enzyme, tomato silage plus sunflower oil	[10–12]
Increased feed intake	Exogenous fibrolytic enzyme (cellulase), flaxseed oil, *Moringa oleifera*	[16–19]
Increased pH/alleviate ruminal acidosis	*Andrographis paniculata*, cereal grains treated with citric acid	[20–22]
Improved digestibility	Exogenous fibrolytic enzyme (cellulase), exogenous fibrolytic enzyme, flaxseed oil, fungal (*Lentinus sajor-caju*) treated oil palm fronds, Lemongrass, *Moringa oleifera*, rosemary, spent mushroom (*Cordyceps militaris*)	[11,16–19,23–25]
Improved volatile fatty acid profiles	Fungal (*Lentinus sajor-caju*) treated oil palm fronds, *Andrographis paniculata*, Fumaric acid	[9,20,21,23]
Improved blood metabolites	Spent mushroom (*Cordyceps militaris*)	[24]
Milk		
Increased milk production	Exogenous fibrolytic enzyme (cellulase), flaxseed oil, humic acid, lemongrass, *Moringa oleifera*, rosemary, *Ziziphus mauritiana*, Sunflower seed, Sunflower seeds oil	[16–19,25,28–30]
Improve milk quality	Exogenous fibrolytic enzyme (cellulase), humic acid, olive oil by-products plus sunflower oil, organic selenium, purple corn stover, tomato silage plus sunflower oil	[10,19,27,30,31]
High milk fat content	Cottonseed cake, *Grewia oppositifolia*, lemongrass, rosemary, sunflower seed, sunflower seeds oil	[25,28,29]
Enhanced Conjugated linoleic acid in milk fat	Canola oil, flaxseed oil, lemongrass, *Moringa oleifera*, pistachio hulls, pomegranate seed pulp, rosemary, tomato pomace, soybean, sunflower seed, sunflower seeds oil	[16–18,25,29,33,34]
Enhanced *Vaccenic* (*t*11–18:1) contents in milk fat	Fish oil, pistachio hulls, pomegranate seed pulp, tomato pomace	[26,33]
Increased total unsaturated fatty acids	Flaxseed oil, hydrolysable tannins, lemongrass, rapeseed oil, rosemary, sunflower seed, sunflower seeds oil	[18,25,29,32]
Others		
Methane mitigation	Fumaric acid, tannin	[9,55]
Improved meat	*Andrographis paniculata*, organic selenium	[20,21,27]

**Table 2:** List of prebiotics and their functions

Prebiotics	Composition	Function	Reference
Cellooligosaccharide	Glucose with beta-1–4 linkages	Modulate the intestinal bacterial community of calves	[56]
Fructooligosaccharides	Spray-dried bovine serum	Reduced the incidence and severity of enteric disease; growth performance	[57,58]
Galactosyl lactose	Trisaccharide (galactose plus lactose); enzymatic treatment of whey with beta-galactosidase	Milk replacer; growth performance	[58,59]
Mannan oligosaccharides	Complex mannose sugars	Block colonization of pathogens in the digestive tract; growth performance	[58]
Pectin oligosaccharides	Pectins (depolymerization of suitable raw materials or purified pectins by partial enzymatic hydrolysis)	Protection of colonic cells against Shiga toxins, Prevention of the adhesion of uropathogenic microorganisms	[60]
Xylooligosaccharides	Lignocellulosic biomass (enzymatic or chemical process from xylan)	Stimulation of beneficial gut microflora, reduction of blood glucose and cholesterol, reduced pro-carcinogenic enzymes in gastrointestinal tract, enhanced mineral absorption from large intestine and immune-stimulation.	[61]
